# Listerine and Lithiated Hydrangea

**Published:** 1884-05

**Authors:** 


### EDITORIAL NOTES.

DR. FOLWELL, who occupied Dr. Hill's house on Delaware avenue, has removed his office and residence to No. 475 Virginia street.

The Buffalo Medical and Surgical Dispensary has removed from Washington street to the "Emergency Hospital," corner South Division and Michigan streets.

We are glad to note the return of Dr. John D. Hill to the city, after a year's absence in Europe His many friends and patients will welcome his return most heartily.

Listerine and Lithiated Hydrangea.—The Buffalo Dispensary was the recipient of a liberal donation of this preparation from the makers. We learn from the professor of surgery that as regards Listerine, its use has but confirmed the high opinion which he has for years had of its value. Lithiated Hydrangea has been used with excellent results in cases of vesical irritation and to correct a uric acid diathesis. The combination of the salts of Lithia with Hydrangea is a happy one, and we are persuaded that in these cases we get more satisfactory results from the use of Lambert's Lithiated Hydrangea than we do from any other preparation.

